# From Gut to Heart: A Case Report of Infectious Endocarditis Stemming From Cholecystitis-Induced Enterococcus faecium Bacteremia

**DOI:** 10.7759/cureus.58683

**Published:** 2024-04-21

**Authors:** Alejandro Martinez-Esteban, Natalia M Barron-Cervantes, Sofia Peña-Solorzano, Jorge-Daniel Sierra-Lara, Carlos Torruco-Sotelo, Regina Faes-Petersen, Alejandro D. G. Gidi, Eduardo Villegas-Tovar

**Affiliations:** 1 General and Gastrointestinal Surgery Service, Fundación Clínica Medica Sur, Mexico City, MEX; 2 General and Gastrointestinal Surgery Service, Fundación Clinica Médica Sur, Mexico City, MEX; 3 Internal Medicine, Fundacion Clinica Medica Sur, Mexico City, MEX; 4 Coronary Care, Fundación Clínica Medica Sur, Mexico City, MEX; 5 Critical Care, Fundación Clínica Medica Sur, Mexico City, MEX; 6 General Surgery, Fundación Clínica Medica Sur, Mexico City, MEX; 7 General and Gastrointestinal Surgery, Fundación Clínica Medica Sur, Mexico City, MEX; 8 General Surgery, Fundación Clínica Médica Sur, Mexico City, MEX

**Keywords:** aortic valve replacement, cervical infection, enterococcus faecium, cholecystitis, infectious endocarditis, bacteremia

## Abstract

Systemic infections are not always going to present as we expect. The study of bacteremia and febrile syndrome represents one of the most important diagnostic challenges nowadays. This case demonstrates the importance of a multidisciplinary approach and finding a common point that explains all the patient's symptoms, no matter how disconnected they may seem. Here, we present the case of a patient where multiple treatments were performed to manage recurrent infective endocarditis due to *Enterococcus faecium* but the cause of this persistence was never found despite surgical management. With only a few cases reported in literature involving this pathogen, it is of great importance to emphasize how searching for a natural reservoir, such as the gallbladder, for this pathogen helped solve the diagnostic mystery that this patient represented. Here, we present how the culture of biological materials, such as the aortic valve replacement, as well as blood cultures, made it possible to identify the etiological agent associated with the pathology and, in turn, find the cause of recurrent bacteremia.

## Introduction

Recurrent endocarditis that does not improve despite a valve change needs to be further studied, looking for an infectious focus that is causing persistent bacteremia. Among the most common causes of bacteremia are urinary infections, the use of intravenous (IV) catheters or drugs, intra-abdominal infections, and cholangitis [1]. Bacteremia is most commonly seen in cases of cholangitis, however, bacteremia in the presence of cholecystitis is not impossible. According to a meta-analysis published in the Scandinavian Journal of Gastroenterology, 7.65% of all confirmed cases of cholecystitis presented with bacterial presence in blood, the most common being Escherichia coli and Klebsiella pneumoniae, corresponding to 87% of all cases. It is important to mention that in this paper, it was concluded that cholecystitis is not often complicated by bacteremia but mostly presented in cases where they presented a previous natural reservoir for that bacteria in the gallbladder [2]. When a patient presents bacteremia, the main risk that we seek to avoid is the presence of secondary endocarditis. The most common cause of endocarditis is Staphylococcus aureus, however, the Enterococci group has been recognized as having significant pathogens associated with it [1]. We present the case of a 70-year-old male patient who was diagnosed with infectious endocarditis and managed by a surgical approach less than a year before and is now presenting to the emergency room (ER) with a fever that does not improve with oral antipyretics in a first-level private surgical center in Mexico City. This case is presented to further expand the knowledge about systemic infection secondary to cholecystitis, as well as highlighting the importance of a multidisciplinary approach including the internal medicine and general surgery team in patients with febrile syndrome, especially with this patient's history.

## Case presentation

**Figure 1:**
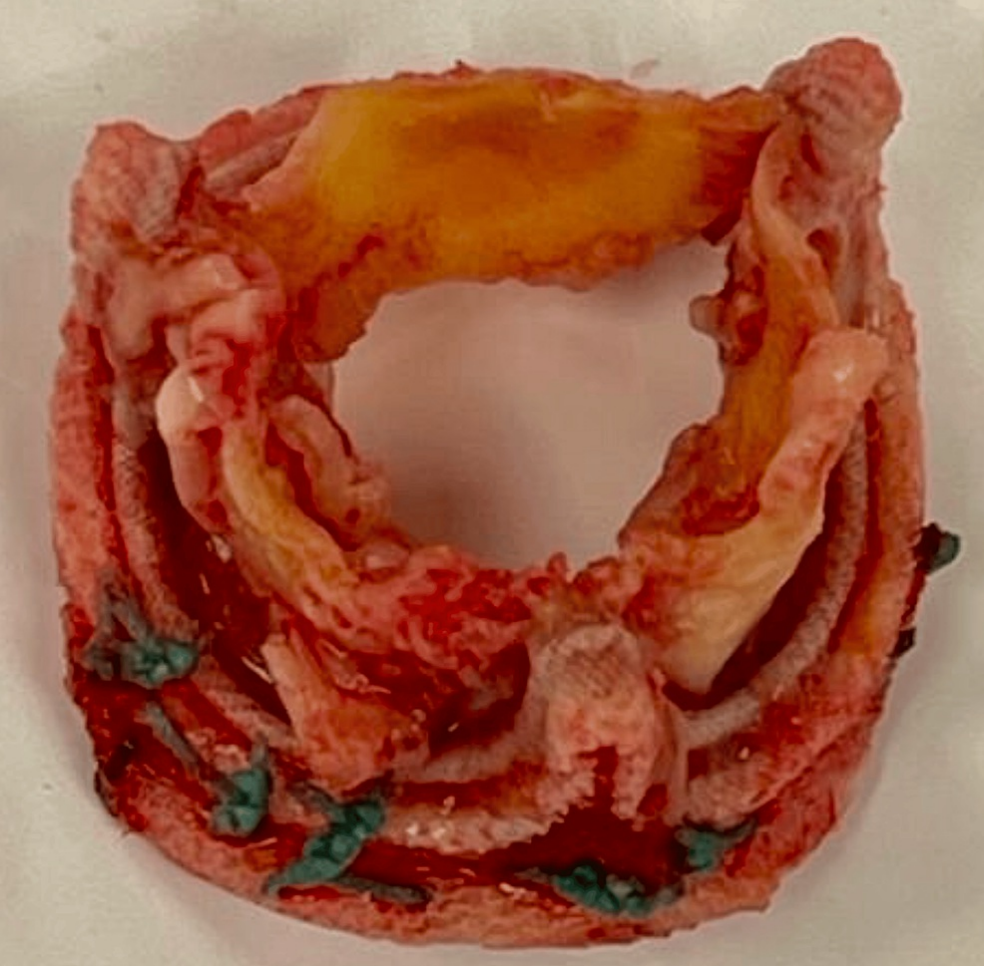
Aortic valve replaced during the second valvular replacement surgery Biological aortic valve prosthesis filled with infectious vegetations positive for Enterococcus faecium

A 70-year-old Latin American man presented to the ER with a quantified fever of up to 38.3°C that did not improve with oral antipyretics. As relevant past medical history, this patient was diagnosed with diabetes type 2 more than 10 years ago, with no complications presented to date. As well, he underwent two aortic valve replacement procedures with biological prosthesis. The first surgery happened two years before the current condition and the second one almost one year later because of two different episodes of bacteremia associated with endocarditis due to Enterococcus faecium complicated with pericarditis. During the second valvular replacement surgery, a biological aortic valve prosthesis filled with infectious vegetations positive for Enterococcus faecium was found (Figure 1). Also, it is important to mention that chronic antibiotic management before surgery was based on levofloxacin, ceftriaxone, and linezolid for five months, and due to there being no response to this treatment, it was decided to perform the second prosthetic replacement. During this last hospitalization, the patient underwent urine, blood, and fecal cultures. The only culture that turned out positive was the blood culture for Enterococcus faecium. Both the urine and fecal cultures were negative. Also, the patient never presented symptomatology suggestive of a urinary or gastrointestinal infective focus.

Because of his past medical history, it was decided to approach it as a febrile syndrome under study. Upon physical examination, no abnormality was found, no cardiac murmurs or arrhythmias were presented, vital signs were within normal limits (WNL), and no abdominal pain was presented. During his stay in the ER, general laboratories were taken. These laboratories showed WHO grade II hypochromic microcytic anemia, mild thrombocytopenia, lymphopenia with the presence of atypical lymphocytes, asymptomatic mild hyponatremia, hypochloremia, hypocalcemia, hypomagnesemia, elevated C-reactive protein (CRP), hypoproteinemia with slightly elevated globulins, alterations in liver function tests presenting an R Factor of 0.1 with a cholestatic pattern and data of iron deficiency (Table 1).

**Table 1:** Laboratory tests performed in the ER Laboratory tests included complete blood count (CBC), blood chemistry (BC), clotting times, hepatic profile, and iron profile

Parameter	Value	Reference values
Hemoglobin	8.5 g/dL	12 - 18 g/dL
Medium Corpuscular Volume	77.7 fl	80 – 100 fl
Mean Corpuscular Hemoglobin	25.8 pg	23 - 31 pg
Hematocrit	25.5 %	36 - 48%
Platelets	147.5 x 10^3^/uL	150 - 450 x 10^3^/uL
Leukocyte Count	5.94 x 10^3^/uL	4.5 - 11 x 10^3^/uL
Absolute Neutrophils	4.4 x 10^3^/uL	2.5 - 7 x 10^3^/uL
Absolute Lymphocytes	1.2 x 10^3^/uL	1 - 4.8 x 10^3^/uL
Prothrombin Time (PT)	11.2 seconds	11 - 13.5 seconds
INR	1.05	1 - 1.1
Partial Thromboplastin Time (PTT)	34.4 seconds	25 - 35 seconds
Serum Glucose	106 mg/dL	70 - 100 mg/dL
Blood Urea Nitrogen (BUN)	21.2 mg/dL	7 - 20 mg/dL
Urea	45.4 mg/dL	5 - 20 mg/dL
Serum Creatinine	0.71 mg/dL	0.6 - 1.1 mg/dL
Serum Sodium	132 mEq/L	135 - 145 mEq/L
Serum Potassium	3.8 mEq/L	3.6 - 5.2 mEq/L
Serum Chlorum	100 mEq/L	96 - 106 mEq/L
Serum Calcium	8.6 mg/dL	8.5 - 10.5 mg/dL
Serum Phosphorus	3.5 mg/dL	2.8 - 4.5 mg/dL
Serum Magnesium	1.7 mg/dL	1.7 - 2.2 mg/dL
Total Serum Proteins	6.4 g/dL	6 - 8 g/dL
Albumin	2.4 g/dL	3.4 - 5.4 g/dL
Globulines	4 g/dL	2 - 3.5 g/dL
Total Bilirubin	0.95 mg/dL	1 - 1.2 mg/dL
Indirect Bilirubin	0.40 mg/dL	0.2 - 1.2 mg/dL
Direct Bilirubin	0.54 mg/dL	0 - 0.35 mg/dL
Glutamic Pyruvic Transaminase (GPT)	22 U/L	4 - 36 U/L
Glutamic-Oxaloacetic Transaminase (GOT)	29 U/L	5 - 40 U/L
Gamma-Glutamyl Transferase (GGT)	372 U/L	0 - 30 U/L
Alkaline Phosphatase (ALP)	498 U/L	44 - 147 U/L
C-reactive Protein (CRP)	228 mg/dL	<0.3 mg/dL
Lactate Dehydrogenase (LDH)	206 U/L	140 - 280 U/L
Serum Iron	18 μmol/L	80 - 180 μmol/L
Transferrin	145	200 - 360 mg/dL
Transferrin Saturation Percentage	8.7 %	15 - 50%

**Figure 2:**
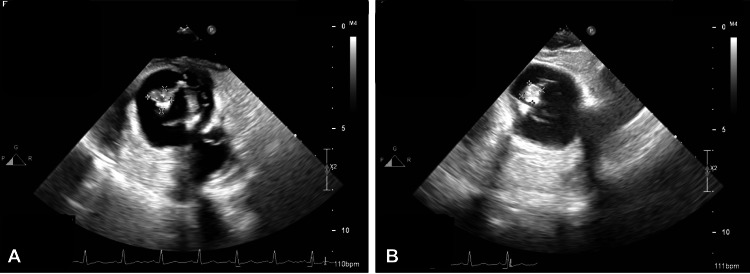
Transesophageal echocardiogram Image A: vegetation on the prosthetic valve; Image B: abscess in the mitral-aortic continuity

Because of his altered liver function profile, especially because of the cholestatic pattern presented, and the febrile syndrome, the conclusion was reached to hospitalize the patient for close monitoring in the Intermediate Therapy Unit (ITU) to complete the diagnostic approach and search for a current infectious focus. Analgesic management was started with acetaminophen 1 gram IV every 8 hours, dexketoprofen 25 mg per oral (PO) every 12 hours, and pregabalin 75 mg PO every 12 hours. Upon his arrival at the ITU, cold blood cultures were requested and the growth of Enterococcus faecium was reported, so a transesophageal echocardiogram (TEE) was performed. The TEE reported findings compatible with probable vegetation on the prosthetic valve and an abscess in mitral-aortic continuity was described (Figure 2).

The diagnosis of infectious endocarditis was made. Therefore, the decision was made to start empiric broad-coverage antibiotic treatment with ertapenem IV 1 gram every 24 hours, gentamicin IV 60 mg every 8 hours, and vancomycin IV 1 gram every 12 hours. This decision was made by the infectology team based on the current antibiotic regimen recommended for acute infectious endocarditis in patients with a previous valvular replacement, composed of six weeks of oxacillin + six weeks of rifampin + two weeks of gentamicin; if oxacillin is not available, as in Mexico, vancomycin can be used, which is what happened in this case [3]. The change of rifampicin to ertapenem was based on the idea that the patient presented with recurrent endocarditis, this being his third episode, by a gram-positive Enterococcus. However, the infectology team wanted to cover other possible options in case these presented later in the final blood cultures, including methicillin-susceptible Staphylococcus aureus, the most common cause of infective endocarditis in prosthetic valves, and Enterobacteriaceae, both covered by ertapenem [4].

**Figure 3:**
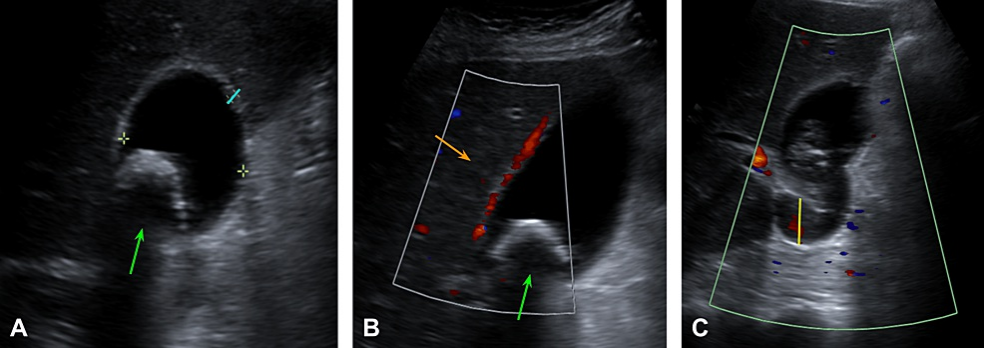
Liver and bile duct ultrasound Image A: Gallbladder lithiasis with a 2.8 cm stone (green arrow) and a 10 x 4 cm gallbladder with thickening of the walls up to 2.6 mm (blue line). Image B: Gallbladder lithiasis (green arrow) and gallbladder edema (orange arrow). Image C: Coledochus diameter of 6.6 mm (yellow line).

Due to the history of this being his third episode of infective endocarditis combined with negative urine and fecal cultures with no symptomatology suggestive of a gastrointestinal or urinary focus, and a cholestatic pattern presented in the initial laboratories taken, it was decided to perform a liver and bile duct ultrasound (US) to rule out an infective focus in the gallbladder or bile ducts. This US reported gallbladder lithiasis with a 2.8 cm stone with rear acoustic shadow, 10 x 4 cm gallbladder with thickening of the walls up to 2.6 mm with edema, and choledochal diameter of 6.6 mm (Figure 3).

**Figure 4:**
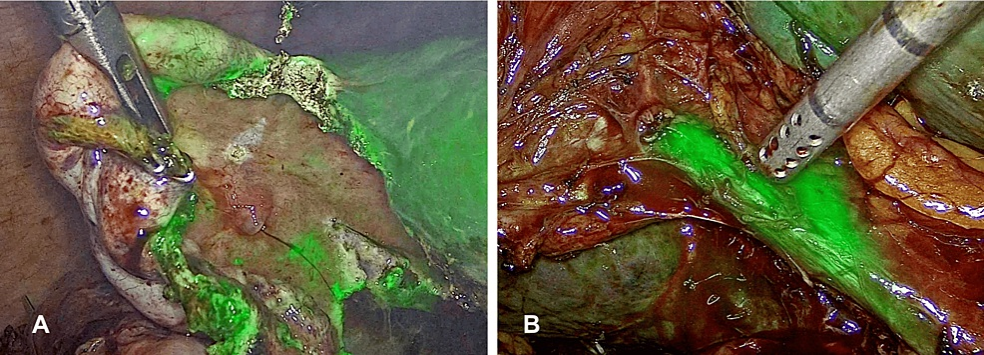
Laparoscopic cholecystectomy with a cholangiography with indocyanine green Image A: Laparoscopic grasper generating traction on the gallbladder after the placement of indocyanine green. Image B: Identification of the cystic duct and common bile duct by near-infrared fluorescent cholangiography with indocyanine green at the level of the hepatocystic triangle.

Based on this, acute stone cholecystitis was diagnosed and the patient was scheduled for laparoscopic cholecystectomy with a cholangiography with indocyanine green. During surgery, a 10 x 4 cm tense, edematous, and erythematous gallbladder with reliable walls and a thickened cystic duct was found. The dissection was carried out to allow Strasberg's critical view to be delimited. Subsequently, using infrared fluorescent cholangiography with indocyanine green, the most important biliary structures, such as the cystic duct and common bile duct, were identified, and the hepatocystic triangle was located (Figure 4).

**Figure 5:**
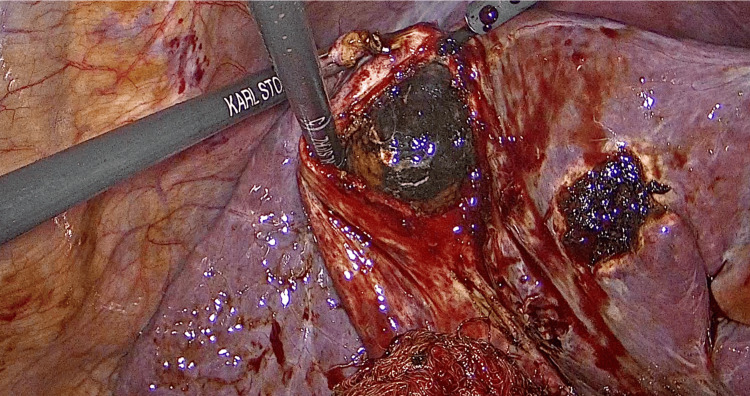
Open gallbladder seen during laparoscopic cholecystectomy Open gallbladder presenting a 3 cm, brown stone of probable infectious origin

Inside the gallbladder, a 3 cm, brown stone of probable infectious origin was found (Figure 5). After surgery, the presence of Enterococcus faecium in the gallbladder was determined through cultures of the bile. As the only pathogen detected in blood and bile cultures was Enterococcus faecium, the infectology team decided to suspend treatment with ertapenem and only finish treatment with vancomycin and gentamicin as previously established. Based on the cultures of the native aortic valve, which were positive for Enterococcus faecium, the previous valve replacement being positive for the same bacteria, and the fact that the blood and bile cultures were equally positive for this agent, it was concluded that the recurrence of endocarditis, concomitant with persistent bacteremia attributed to Enterococcus faecium, originated from the biliary reservoir detected. After the postoperative period of the cholecystectomy and after completing the previously mentioned antibiotic regimen, based on two weeks of gentamicin and six weeks of vancomycin, a new aortic valve replacement and lavage and drainage of the abscess in mitral-aortic continuity were scheduled. After his post-surgical recovery, he was discharged from the cardiothoracic surgery service and the general surgery service, with adequate control of previous symptoms. The last blood cultures performed before discharge were negative and the patient no longer had fever spikes. He is currently healthy, completely asymptomatic, and without new episodes of bacteremia since said hospitalization, one year ago. It is important to emphasize that this was a patient with recurrent positive cultures for Enterococcus faecium and since the cholecystectomy, the patient has not presented new episodes of bacteremia or symptoms suggestive of it.

## Discussion

Infectious endocarditis is a disease associated with high morbidity and mortality rates. The most common etiology associated with this condition is Staphylococcus aureus, with up to 30% of all cases. Then, the Streptococci species are the second most common cause. Fungal endocarditis is rare, with only 1% of all cases, however, it is important to mention, as it is associated with fatal outcomes associated with systemic Candida or Aspergillus infection in immunocompromised patients [5]. The major risk factors presented are vascular catheterization, hemodialysis, hospitalization, and non-cardiac surgery; in these cases, the valve was previously healthy before the infection and the most common cause is Staphylococcus aureus. Whereas, when the infection happens on previously disease valves, such as in cases where vascular devices or prosthetic valves were placed, the most common cause is Staphylococcus epidermidis [6].

The development of this condition requires a previous endocardial injury followed by a period of bacteremia. The main theory is that endocardial damage may emerge as a result of turbulent flow or previously diseased valves, which is why cases of prior cardiac surgery are at high risk of developing infectious endocarditis under the presence of persistent bacteremia [7]. For many decades, the diagnosis has been based on the Duke Criteria and later on with the Modified Duke Criteria, which is based on the presence of two major criteria, one major and three minor or five major criteria. Major criteria include the confirmation of bacteremia, including two separate blood cultures positive to a typical organism and sonographic evidence of endocardial involvement, the most sensitive method being TEE. Antibiotic treatment in prosthetic valve cases should be based on gentamicin, vancomycin, and rifampicin for at least two to six weeks [8]. Surgical intervention is indicated in cases of acute heart failure, extensive infection with local complications, or recurrent arterial embolization [9]. In this case, the patient underwent valvular replacement because of the abscess in mitral-aortic continuity presented.

Acute cholecystitis results from the obstruction of the cystic conduct by an impacted gallstone, this consequently shows as an edematous wall and gallbladder distension. The incidence of culture-positive bile in patients with cholecystitis is 35% to 65%, being the most commonly isolated pathogens are Escherichia coli and Klebsiella pneumoniae [10]. The typical clinical presentation of cholecystitis includes right upper quadrant pain, fever, and nausea. However, there are some cases where the cystic conduct isn’t completely obstructed, thus it may present asymptomatic, in this case, the correct medical term is cholelithiasis, not cholecystitis. The preferred image study used to establish the diagnosis is liver and bile duct US, with a sensitivity of 81% and a specificity of 83%. However, it is important to mention that when no definitive diagnosis can be established, hepatobiliary scintigraphy is the preferred diagnostic test [11].

The gallbladder can be a natural reservoir for some pathogens, as this case presents. Particularly in this report, the patient had negative urine and fecal cultures and no symptomatology suggestive of a gastrointestinal or urological infective focus, this fact combined with the cholestatic pattern presented upon admission was key in determining the use of image studies to try to locate an infective focus in the bile ducts or gallbladder. Long-term invasion into the gallbladder is consistent with a biofilm/related disease, they typically adhere to each other and are encased in the extracellular matrix [12]. Biofilms are usually presented on the surface of cholesterol gallstones in vitro and in vivo [13]. In cases where there is a chronic infection associated with this reservoir, the only treatment option is a cholecystectomy [12]. All of this information is critical in understanding the case presented here, as it has been proven that Enterococcus faecium, specifically the subtype CC17, has an important role in biofilm formation, thus allowing the patient’s gallbladder to be a reservoir for this pathogen [14].

This report presents a case where the patient had proven bacteremia and endocarditis by Enterococcus faecium, a gram-positive enterococci usually presented in the nosocomial setting. Nowadays, the current antibiotic regimen recommended for acute infectious endocarditis in patients with a previous valvular replacement is composed of six weeks of oxacillin + six weeks of rifampin + two weeks of gentamicin; If oxacillin is not available, as it is in Mexico, vancomycin can be used [3]. The use of empiric ertapenem was made by the infectology team, as they wanted to cover other possible options in case these presented later in the final blood cultures, including methicillin-susceptible Staphylococcus aureus, the most common cause of infective endocarditis in prosthetic valves [15] and Enterobacteriaceae, both cover by ertapenem [4]. As mentioned in the case presentation, when the final blood culture report turned out to be positive only for Enterococcus faecium and no other pathogens were detected, the decision to suspend ertapenem was made.

## Conclusions

This case underscores the significant importance of adopting an interdisciplinary approach when confronted with cases characterized by multifactorial etiology for concurrent patient symptoms. The persistent occurrence of endocarditis despite surgical intervention highlighted the presence of an underlying source of infection within the patient, perpetuating subsequent instances of valve replacement infection. Deviating from conventional antibiotic management protocols, the treatment strategy in this instance was tailored to accommodate the antibiotic availability in our region while considering the clinical profile of a patient with recurrent infections and heightened susceptibility to secondary pathogens. This case serves as a poignant reminder that comprehensive patient care necessitates the collaboration of multiple medical specialties, each contributing their expertise toward optimizing patient outcomes. Furthermore, the decision to proceed with cholecystectomy surgery represented a pivotal step toward enhancing the overall health status of the patient, thereby not only mitigating mortality risk but also ameliorating morbidity concerns.
